# Southern Ocean pteropods at risk from ocean warming and acidification

**DOI:** 10.1007/s00227-017-3261-3

**Published:** 2017-11-10

**Authors:** Jessie Gardner, Clara Manno, Dorothee C. E. Bakker, Victoria L. Peck, Geraint A. Tarling

**Affiliations:** 1 British Antarctic Survey, Natural Environment Research Council, Cambridge, UK; 2 Centre for Ocean and Atmospheric Sciences, School of Environmental Sciences, University of East Anglia, Norwich, UK

## Abstract

Early life stages of marine calcifiers are particularly vulnerable to climate change. In the Southern Ocean aragonite undersaturation events and areas of rapid warming already occur and are predicted to increase in extent. Here, we present the first study to successfully hatch the polar pteropod *Limacina helicina antarctica* and observe the potential impact of exposure to increased temperature and aragonite undersaturation resulting from ocean acidification (OA) on the early life stage survival and shell morphology. High larval mortality (up to 39%) was observed in individuals exposed to perturbed conditions. Warming and OA induced extensive shell malformation and dissolution, respectively, increasing shell fragility. Furthermore, shell growth decreased, with variation between treatments and exposure time. Our results demonstrate that short-term exposure through passing through hotspots of OA and warming poses a serious threat to pteropod recruitment and long-term population viability.

**Electronic supplementary material:**

The online version of this article (10.1007/s00227-017-3261-3) contains supplementary material, which is available to authorized users.

## Introduction

A multitude of concurrent drivers poses a pernicious, global threat to marine ecosystems and their services (Bijma et al. 2013). With increases in temperature of 0.6–2.0 °C, decreases in pH of 0.1–0.4 units and shoaling of the carbonate compensation depth forecasted to occur by 2100 (RCP 2.6–8.5), ocean acidification (OA), and warming pose an acute threat to marine organisms (IPCC 2013; Heinze et al. 2015). Understanding the impact of these environmental perturbations on marine biota remains a major challenge (Kroeker et al. 2013), since a range of intra- and inter-specific responses to multi-stressors have been observed (Wernberg et al. 2012).

Early life history stages are suspected to be especially vulnerable to environmental change (Dupont and Thorndyke 2009), although large variations in tolerance have been observed (Foo and Byrne 2017). Viability and survival of these stages are vital for successful recruitment and long-term population stability. Furthermore, early exposure to environmental stressors could alter vulnerability of later developmental stages through latent effects (such as mortality and shell size), adding to the overall impact (Kroeker et al. 2013; Suckling et al. 2014).

High latitude ecosystems are expected to experience OA first due to seasonal amplification, freshening, and colder temperatures enhancing CO_2_ solubility (Doney et al. 2009; Fabry et al. 2009). Such processes alter the ratio of dissolved inorganic carbon (DIC) and total alkalinity (TA) to values where calcium carbonate becomes susceptible to dissolution. This poses a significant threat to polar marine organisms that form calcium carbonate shells, skeletons, or internal structures (Mostofa et al. 2016). In the Southern Ocean carbonate undersaturation events have already been observed (Bednaršek et al. 2012a) and are predicted to occur more frequently over the coming decades (McNeil and Matear 2008).

Thecosome pteropods (holoplanktonic gastropods) can dominate high latitude zooplankton communities (Hunt et al. 2008). In polar regions, shelled pteropods are one of the major components within food webs, acting as a food source of carnivorous zooplankton, fishes, and a number of higher predators (Falk-Petersen and Sargent 2001). Furthermore, polar pteropods contribute significantly to carbon and carbonate export to the deep ocean through the sinking of dead individuals and faecal pellets (Manno et al. 2007, 2009). *Limacina helicina* are true polar pteropods with its two species, *Limacina helicina antarctica* and *Limacina helicina helicina* occurring within the Antarctic and Arctic, respectively (Hunt et al. 2010). They are considered sentinels of OA, since their shells consist of aragonite, a relatively soluble polymorph of calcium carbonate (Mucci 1983; Bednaršek et al. 2014). In the Southern Ocean, *L. helicina antarctica* reside within the surface ocean where aragonite undersaturation events and ‘hotspots’ of rapid warming have already been identified and are predicted to become more frequent (McNeil and Matear 2008; Gutt et al. 2015; Vaughan et al. 2003). This is of particular concern in the northern Scotia Sea region, since it has the largest measured seasonal cycle of surface ocean CO_2_ in the Southern Ocean (Jones et al. 2012, 2015) as well as upwelling events of CO_2_ enriched deep water to the surface and hotspots of warming (Bednaršek et al. 2012a; Whitehouse et al. 2008; Gille 2002).

Shell dissolution of juvenile *L. helicina antarctica* has already been reported in natural populations within the Scotia Sea (Bednaršek et al. 2012a), while numerous incubation experiments under predicted OA levels of polar pteropod juveniles and adults suggest a range of other negative physiological responses (Comeau et al. 2009; Lischka et al. 2011; Manno et al. 2012; Peck et al. 2016a, b; Seibel et al. 2012). Manno et al. (2016) demonstrated that maternal and embryonic exposure of *L. helicina antarctica* to acidified conditions reduced the percentage of eggs developing to later stages by 80%. However, responses to concurrent OA and warming remain unresolved (Bednaršek et al. 2016a, b) particularly with regard multi-stressor responses of larval *L. helicina antarctica.* To date, studies of larval pteropods have focussed on incubating North Atlantic and Mediterranean species with *Limacina retroversa* exhibiting increased mortality (Thabet et al. 2015) and *Cavolinia inflexa* shell malformations (Comeau et al. 2010a) as a result of ocean acidification. Due to the key ecological and biogeochemical roles of *L. helicina antarctica* in polar regions, alteration of larval shell morphology alongside reduced recruitment to adulthood could have major implications on the Southern Ocean ecosystem.

In this study, we successfully hatched cultivated polar shelled pteropods (*L. helicina antarctica*) and used these to examine responses to the singular and combined impact of acidification and warming on posthatch shell development, morphology, and survival. These short-term incubations aim to simulate the experience of larvae (veliger stage) to variations in their environment as a result of present day and predicted heterogeneity in warm and acidified waters in the Southern Ocean (McNeil and Matear 2008; Gutt et al. 2015; Vaughan et al. 2003). Larval survivorship and fitness underpin recruitment success and any negative impacts from a high CO_2_ world can ultimately reduce long-term population viability in this region (Przeslawski et al. 2008).

## Methods

*Limacina helicina antarctica* were collected aboard the RRS James Clark Ross (Cruise number JR304) within the Scotia Sea (57°36′20.5″S, 43°40′22.2″W) in November 2014 using a motion-compensated Bongo net (100 and 200 µm mesh sizes), vertically hauled from 200 m. The motion compensation reduced stress on pteropods during collection and avoided mechanical damage to shells. Ambient sea-surface conditions at 10 m were characterised by a sea-surface temperature and salinity of 1.62 and 34.3 °C, respectively, with a TA (total alkalinity) of 2320 µmol/kg and a pH (total scale) of 8.09.

Live adult females were identified following the description of Lalli and Wells (1978) and examined under a light microscope (Olympus SZX16 fitted with a Cannon EOS 60D). Actively swimming individuals with no signs of damage (shell and body) and fully translucent shells were acclimated within filtered seawater (0.22 µm) for 8 h at 1.66 ± 0.03 °C (Spartel incubator with a C-400 circulator unit and an FC-500 in-line cooler, temperature measured every 2 h). After this, individuals that were actively swimming were placed individually within 500 ml incubation jars (non-pyrogenic polystyrene, Corning^®^) filled with filtered seawater and maintained at 1.22 ± 0.41 °C. Jars were stored in darkness and sealed with no headspace to limit CO_2_ exchange and inspected at least every 6 h.

After 9 days, some adults spawned eggs within a 2-h period. These were immediately removed using a wide mouthed Pasteur pipette to avoid egg cannibalism and damage. Mothers were in a good state of health during egg production (i.e., swimming and maintaining fully transparent shells) (Peck et al. 2016a, b). Egg ribbons were placed separately into ambient incubatory conditions within 65 ml jars of filtered seawater (1.02 ± 0.31 °C). Following further 7 days of incubation, veligers emerged simultaneously from four egg ribbons, each having been laid by a different female. These were mixed and randomly transferred into experimental conditions via stretched glass pipettes.

### Experimental design

All incubations took place within a controlled temperature room aboard the RRS James Clark Ross. Posthatch veligers were examined under a light microscope and five actively swimming individuals with fully translucent shells were placed together into each of the 60 incubation jars (65 ml, non-pyrogenic polystyrene, Corning^®^). Fifteen jars were placed into each of the four treatments exemplifying ambient (1.7 °C and pH 8.1), warm (3.5 °C and pH 8.1), acidified (1.7 °C and pH 7.6) or acidified-warm (3.5 °C and pH 7.6) conditions (Table 1) based upon predictions in 2100. All seawater was filtered (0.22 µm), since the current evidence suggests *L. helicina antarctica* spawn in the autumn and overwinter as larvae when food availability is naturally low (Hunt et al. 2008; Lischka and Riebesell 2016). Furthermore, filtration removes the possibility of biological activity altering the carbonate chemistry within the treatment bottles from the target values. To examine the impact of exposure time, three bottles were removed from each treatment every day for 5 days (Supplementary material Figure 1). Each bottle was gently decanted into deep-welled glass petri dishes and veligers were inspected under a light microscope for 5 min each. Those that were actively swimming and/or showed ciliate velum activity were classed as alive. Maximum shell length was measured using a graticule and condition of the larvae noted before preservation, ensuring no secondary preservation effects occurred. All veligers were subsequently rinsed with de-ionised water three times. For preservation, two specimens from each bottle were air dried upon a filter, while the remainder were placed into Eppendorf tubes filled with 70% buffered ethanol.

**Table 1:** Mean (± SD) values of carbonate system parameters determined from water samples from each treatment

Treatment	Temperature (°C)	TA (μmol/kg)	DIC (μmol/kg)	Start pH End pH	*Ω*_ar_	pCO_2_ (μatm)
Adults	1.22 ± 0.41	2293	2140	8.12 ± 0.01	1.12	364
				8.10 ± 0.02		
Ambient	1.71 ± 0.05	2322	2162	8.11 ± 0.01	1.38	342
				8.10 ± 0.01		
Warm	3.50 ± 0.09	2319	2154	8.09 ± 0.01	1.49	343
				8.09 ± 0.01		
Acidified	1.71 ± 0.05	2329	2332	7.60 ± 0.01	0.62	1180
				7.60 ± 0.01		
Acidified-warm	3.50 ± 0.09	2325	2320	7.60 ± 0.01	0.61	1194
				7.60 ± 0.01		

Treatment refers to the target incubation conditions. Temperature was measured every 4 h, while pH (total scale) at the start and end of each incubatory period. Salinity, TA, and DIC were determined from samples taken at the start of the experiment and subsequently used to calculate pCO_2_ (partial pressure of CO_2_) and *Ω*_ar_ (aragonite saturation state) using CO_2_SYS. Salinity was 34.5

Ethanol preserved veligers were dehydrated through a series of ethanol solutions (50, 70, 80, 90, 95, and 100%, 5 min each) to stop shell collapse while dried veligers needed no further preparation. All veligers were subsequently mounted on carbon tape and imaged at 1200× magnification using a variable pressure scanning electron microscope (SEM) (TM3000, Hitachi). Only specimens that were living at the end of the incubation were considered for shell analysis (*n* = 233). Using the SEM, the apical shell surface was inspected for the presence or absence of pitting (deep holes in the shell surface), etching (the partial dissolution of the upper shell surface observed by exposure of the granular or prismatic layer beneath), and malformation (deviation of growth from the expected smooth spiral) (Fig. 4). Since, statistically, there was complete separation in the presence/absence of etching and pitting (for example in etching, presence was either 100 or 0%), this resolution of shell analysis was considered appropriate. The maximum shell diameter was also measured using the SEM and light microscope graticule to approximate shell size over the exposure period.

### Seawater manipulation

A Spartel incubator with a C-400 circulator unit and an FC-500 in-line cooler housed within the ship’s cold room was used to control incubation temperature. Temperature was measured every 4 h throughout the entire incubatory period (PreSens fibox 4).

Seawater pH was manipulated through additions of HCl (hydrochloric acid) and NaHCO_3_ (sodium bicarbonate) calculated by the seacarb software and maintained in a closed system (Lavigne and Gattuso 2010). Gas bubbling and addition of acid/bicarbonate and/or carbonate are considered some of the best methods to mimic ocean acidification (Gattuso et al. 2011). Acid/base addition was used in this instance due to the short incubation period, bottle volumes, and logistical restraints besides minimising the risk of damage to veligers as observed from bubbling (Howes et al. 2014; Gattuso and Lavigne 2009). Although pH may be lower than expected from this technique, we believe that the values are within the range predicted to occur by 2100 (RCP 8.5) in the Southern Ocean (Table 1) (IPCC 2013; Gattuso et al. 2011; Gattuso and Lavigne 2009; SCAR 2009; Schulz et al. 2009; McNeil and Matear 2008). The calculation utilised measurements of pH (total scale) made with a pH electrode (Metrohm 826) and TA through applying a sea-surface salinity (*S*) and temperature (*T*) algorithm based on Lee et al. (2006) and refined through a recent spatially intensive carbonate chemistry survey in this region (M.P. Humphreys, pers. comm.):

1 $${\text{TA}} = 683.41S - 9.139S^{2} - 1.37T - 0.896T^{2} - 10364.16.$$

To determine the impact of the manipulations on the incubation water, we extracted a 250 ml sub-sample of initial incubatory conditions, fixed with mercuric chloride, and stored in a borosilicate bottle for subsequent analysis of TA and DIC. TA was measured by potentiometric titration and DIC by coulometry using a VINDTA (Versatile Instrument for the Determination of Titration Alkalinity, version 3C). Accuracy (TA = 2.5 µmol/kg; DIC = 1.1 µmol/kg) was determined using certified reference materials (Scripps Institution of Oceanography). pH was determined at the start and end of the incubation experiment. Aragonite saturation state was indirectly estimated from TA and DIC values using CO_2_SYS software with the constants of Mehrbach et al. (1973) refitted by Dickson and Millero (1987) and sulphate dissolution constants by Dickson (1990). Carbonate system parameters of the incubations are shown in Table 1.

### Statistical analysis

Data were analysed using R (2015). All larvae were considered when estimating mortality between treatment and days (*n* = 300) exposed; however, only larvae that were living at the end of the incubatory period and were not damaged during processing were included within the analysis of shell morphology and size (*n* = 233 where ambient *n* = 66, acidified *n* = 44, warm = 59, acidified-warm = 54). A binomial (logit) generalised linear model (GLM) was used to estimate whether there were any differences in etching, pitting, and malformation presence between treatments and days exposed. A gamma (identity) GLM and a binomial (logit) GLM estimated differences in shell size and mortality between treatments and days of exposure, respectively. Complete separation between treatments for the presence of shell etching and pitting was found; therefore, a Bayesian analysis with non-informative prior assumptions (Gelman et al. 2008) was utilised from the arm package (Gelman and Su 2016). Model selection was informed using the information theoretic approach using the MuMIn package (Barton 2016) to identify the models with delta Akaike information criterion < 4 and the highest Akaike weights (Supplementary material Table 8) alongside comparisons of *R* ^2^ and likelihood ratio tests using the lmtest package (Zeileis and Hothorn 2002). Model validation included checking assumptions of residual normality and Homoscedasticity, overdispersion, autocorrelation, collinearity, and independence. For post hoc analysis, Tukey’s pairwise comparisons were performed using the lsmeans (Lenth 2002). A two-way factorial analysis was also used within the same model frameworks as above to highlight the presence of any interactions between warming, acidification, and exposure time on larval shell morphology, size, and mortality.

Confidence intervals (CIs) for mean mortality *x* were calculated by the following:

2 $${\text{CI}} = x \pm \frac{Z\alpha }{2}\sigma /\surd {\text{n,}}$$

where *n* is the number of living larvae per incubation bottle at the start of the incubation, *σ* the standard deviation, and *Zα*/2 the *Z*-table value for a given α value. Confidence intervals for the mean occurrence of shell malformation, pitting, and etching as well as larval mortality were calculated by the modified Wald method (Agresti and Coull 1998).

## Results

### Change in larval pteropod mortality

**Fig. 1:**
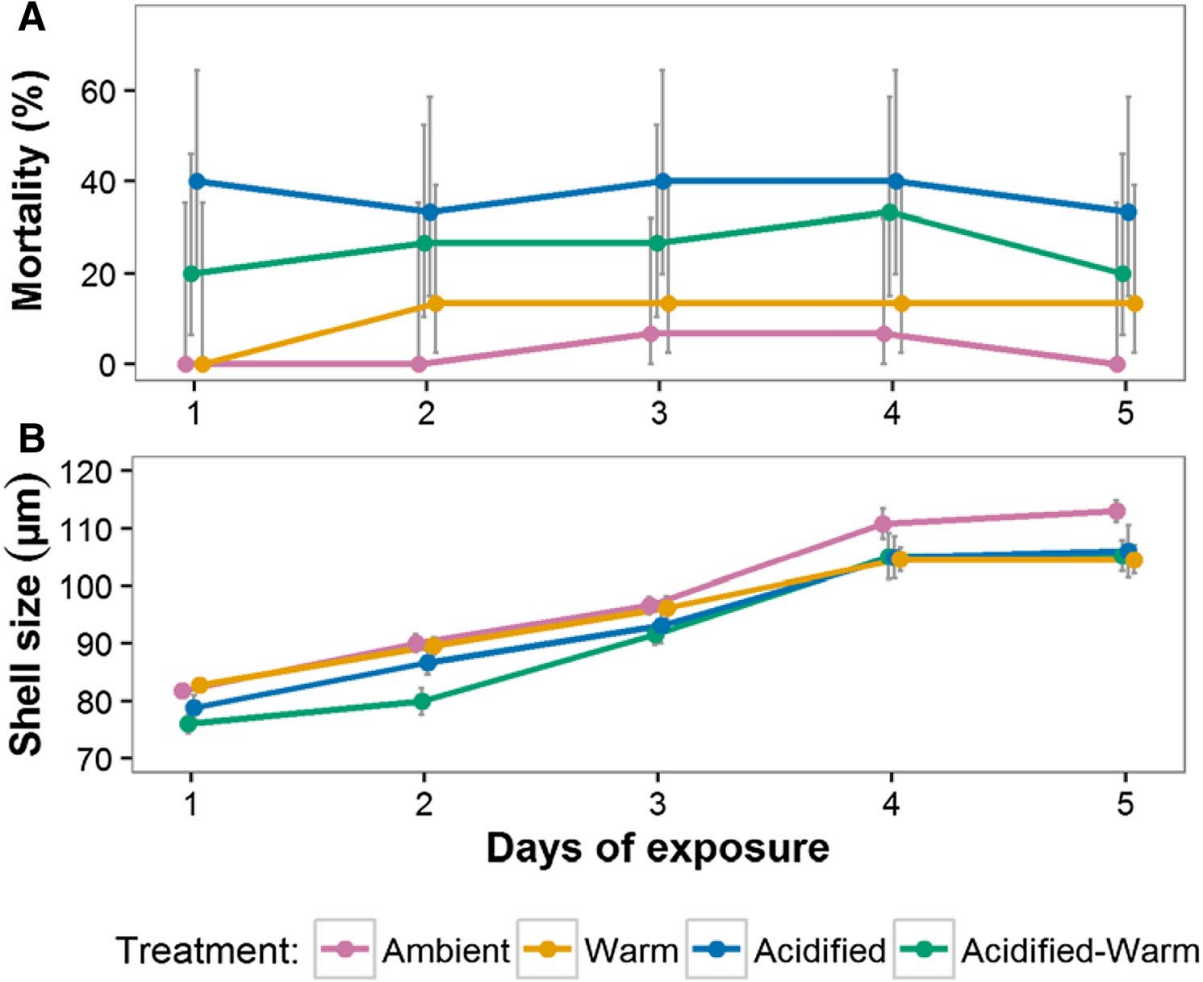
*Limacina helicina antarctica* larval mortality (**a**) and shell size (**b**) over time during incubation under ambient (pink), warm (orange), acidified (blue), and acidified-warm (green) conditions. Only larvae that were alive upon harvesting were included in the analysis of shell size. Bars denote 95% confidence intervals between treatment bottles

Significantly more larval fatalities occurred within warm, acidified, and acidified-warm conditions overall in comparison to ambient treatments (*p* < 0.01) (Fig. 1a). However, the number of fatalities did not change with the amount of time exposed to these conditions (*p* > 0.05) (Supplementary materials Table 1). A post hoc analysis showed that throughout, mortality was significantly higher in acidified conditions (38.7%, *n* = 29) and acidified-warm conditions (25.3%, *n* = 19) compared to that in warm (12%, *n* = 9) and ambient conditions (2.7%, *n* = 2) (*p* < 0.001). Furthermore, a factorial analysis indicated that acidification (*p* < 0.001), rather than warming (*p* > 0.05), increases larval mortality. However, larval mortality significantly increases when warming and acidification are combined (*p* < 0.01) (Supplementary materials Table 2). For a summary of mortalities, see Supplementary materials Table 3.

### Change in larval pteropod shell size

Larvae in all treatments significantly increased their shell size during the incubation (*p* < 0.001). Over the entire exposure time, larvae experiencing acidified (*p* < 0.05) and acidified-warm (*p* < 0.01) conditions were smaller than in ambient and warm conditions, which were similar (*p* > 0.05) (Fig. 1b) (Supplementary materials Table 4). Post hoc analysis indicated that shells incubated in acidified-warm conditions were significantly smaller than those in ambient conditions (*p* > 0.001) but were not different from those in acidified conditions (*p* > 0.05). Furthermore, during the first 3 days of exposure, shell size was similar between ambient, warm, and acidified conditions, but subsequently, shell size was smaller in warm and acidified conditions relative to ambient conditions (*p* < 0.01). The rate of change in shell size was significantly lower in acidified-warm conditions on day 2 (*p* < 0.05), warm conditions on day 4 and 5 (*p* < 0.001), and acidified conditions on day 5 (*p* < 0.05) (Supplementary materials Table 4). This resulted in shell size on day 5 being smaller on exposure to warming (104.5 ± 1.11 µm, *n* = 13), acidified (106.0 ± 2.0 µm, *n* = 10), and acidified-warm conditions (105.2 ± 1.2 µm, *n* = 12) compared to ambient conditions (113.0 ± 0.9 µm, *n* = 14). The factorial analysis indicated that shell growth was primarily reduced by exposure to acidified conditions (*p* < 0.001) rather than warm (*p* > 0.05) with no interaction between them (*p* > 0.05).

### Change in larval pteropod shell morphology

**Fig. 2:**
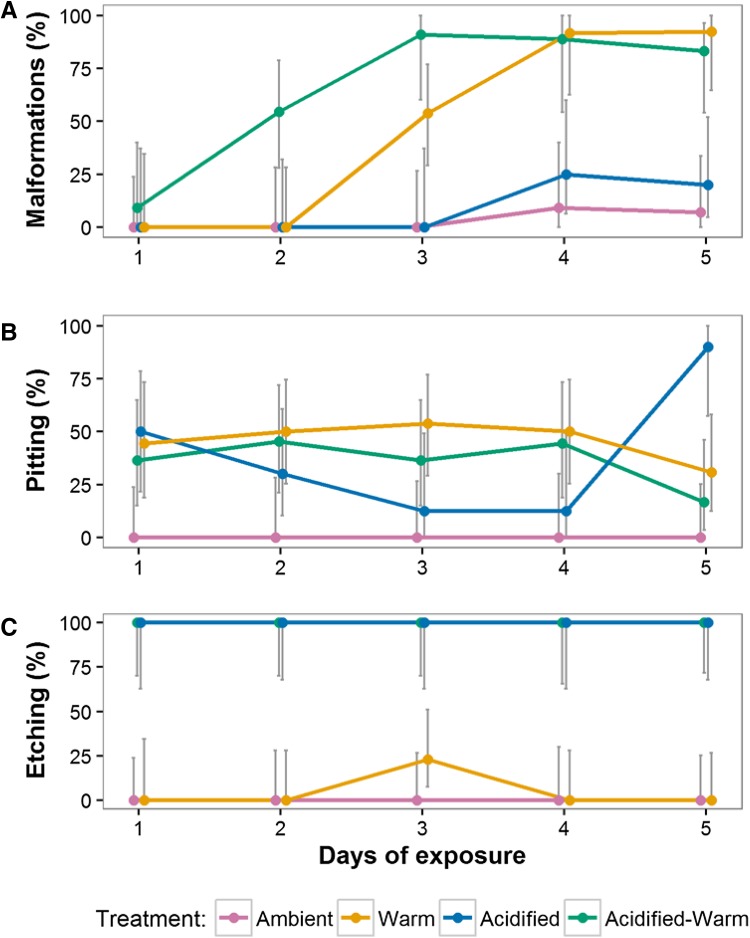
*Limacina helicina antarctica* larval shell condition showing percentage occurrence of **a** malformation, **b** pitting, and **c** etching over time during incubation under ambient (pink), warm (orange), acidified (blue), and acidified-warm (green) conditions. Bars denote 95% confidence intervals between treatment bottles. Only larvae that were alive upon harvesting were included (*n* = 223)

*Malformation* Significantly more larval shell malformations were present in response to warm and acidified-warm conditions compared to ambient and acidified conditions (*p* < 0.0001) (Fig. 2a) (Supplementary material Table 6). Post hoc analysis indicated a similar number of larvae developed malformations on exposure to acidified-warm (62.5%, *n* = 35) and warm conditions (49.2%, *n* = 29) (*p* > 0.05). Likewise, there was no difference in the number of malformations between acidified (9.1%, *n* = 4) and ambient conditions (3%, *n* = 2) (*p* > 0.05). Malformations occurred as a result of exposure to warm conditions (*p* > 0.001) rather than acidification (*p* > 0.05) with no interaction between them (*p* > 0.05) (Supplementary material Table 7). The number of shell malformations was highly dependent on the amount of time that the individuals were exposed to each condition (*p* < 0.0001, *n* = 223), with larval malformation instances significantly increasing after 3 days of exposure (*p* < 0.001). There was an 82% increase in malformation occurrence within acidified-warm conditions after the first 3 days of exposure. Furthermore, the number of malformations gradually increased in warming conditions with none being present on day 1–92% being malformed after 5 days.

*Pitting* Larvae that experienced warm, acidified, and acidified-warm conditions all displayed significantly higher amounts of shell pitting than those incubated in ambient conditions (Fig. 2b) (*p* < 0.001, *n* = 223) (Supplementary material Table 6). Larval shells with the most pitting (47%, *n* = 28) were found in warm conditions, although this was not statistically different from the pitting instances on larval shells exposed to acidified-warm (40%, *n* = 20) or acidified (23%, *n* = 10) conditions (*p* > 0.05). Warming and acidification both increased the instances of pitting; however, the combination of acidified-warm conditions does not increase pitting instances as much as would be expected from an additive or a synergistic response (*p* < 0.01, *n* = 223) (Supplementary material Table 7). The amount of time larvae were exposed to each condition did not alter the number of pitting instances (*p* > 0.05, *n* = 223).

*Etching* There were significantly more cases of shell etching in acidified and acidified-warm conditions compared to ambient and warm conditions (*p* < 0.001, *n* = 223) (Fig. 2c) (Supplementary material Table 6). The presence of etching was attributable to acidification only (*p* < 0.001, *n* = 223) with no effect of exposure to warm conditions or an interaction (*p* > 0.05, *n* = 223) (Supplementary material Table 7). Larvae incubated in ambient and warm conditions exhibited either no or few cases (2.97%, *n* = 3) of etching, respectively, with no significant difference between the conditions (*p* > 0.05). Conversely, after 1 day (24 h) of exposure to acidified and acidified-warm conditions, all larvae had shell etching present, and there was no change in the instances of etching over time (*p* > 0.05, *n* = 223).

### Overall shell morphology

**Fig. 3:**
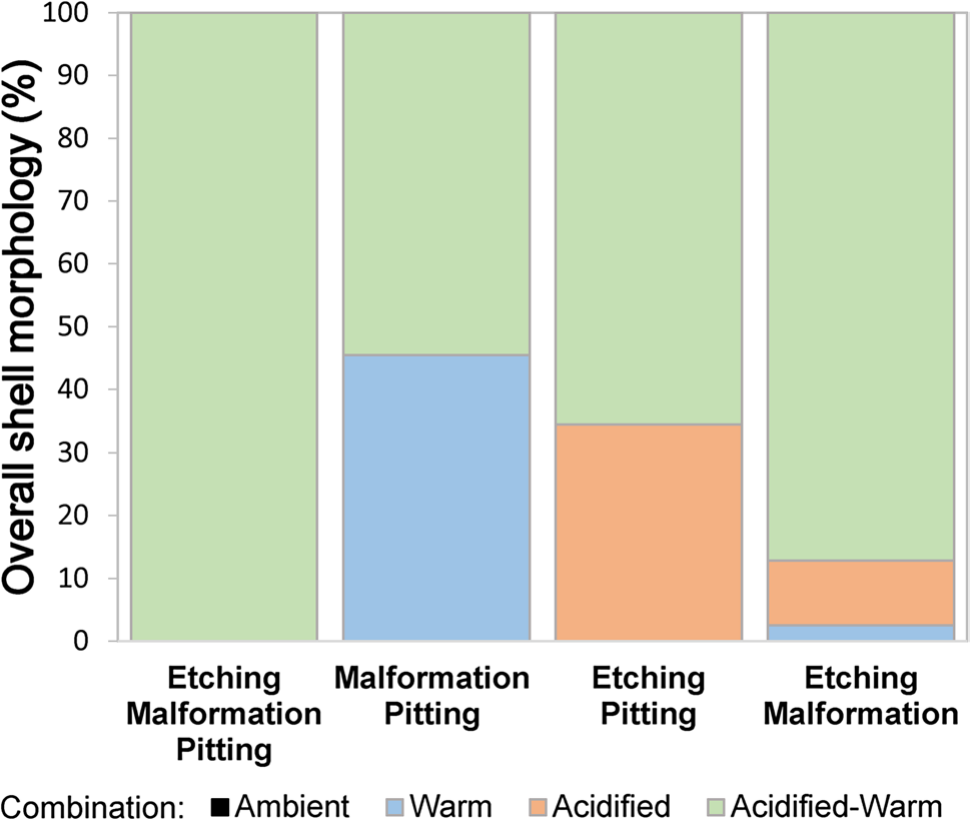
Larval *Limacina helicina antarctica* shell morphology over 5 days of exposure to ambient, warm, acidified, and acidified-warm conditions. Each bar shows the treatments where a combination of different shell morphologies (pitting, malformation, and etching) developed on the same single larval shell. Only larvae that were alive upon harvesting were included (*n* = 223)

**Fig. 4:**
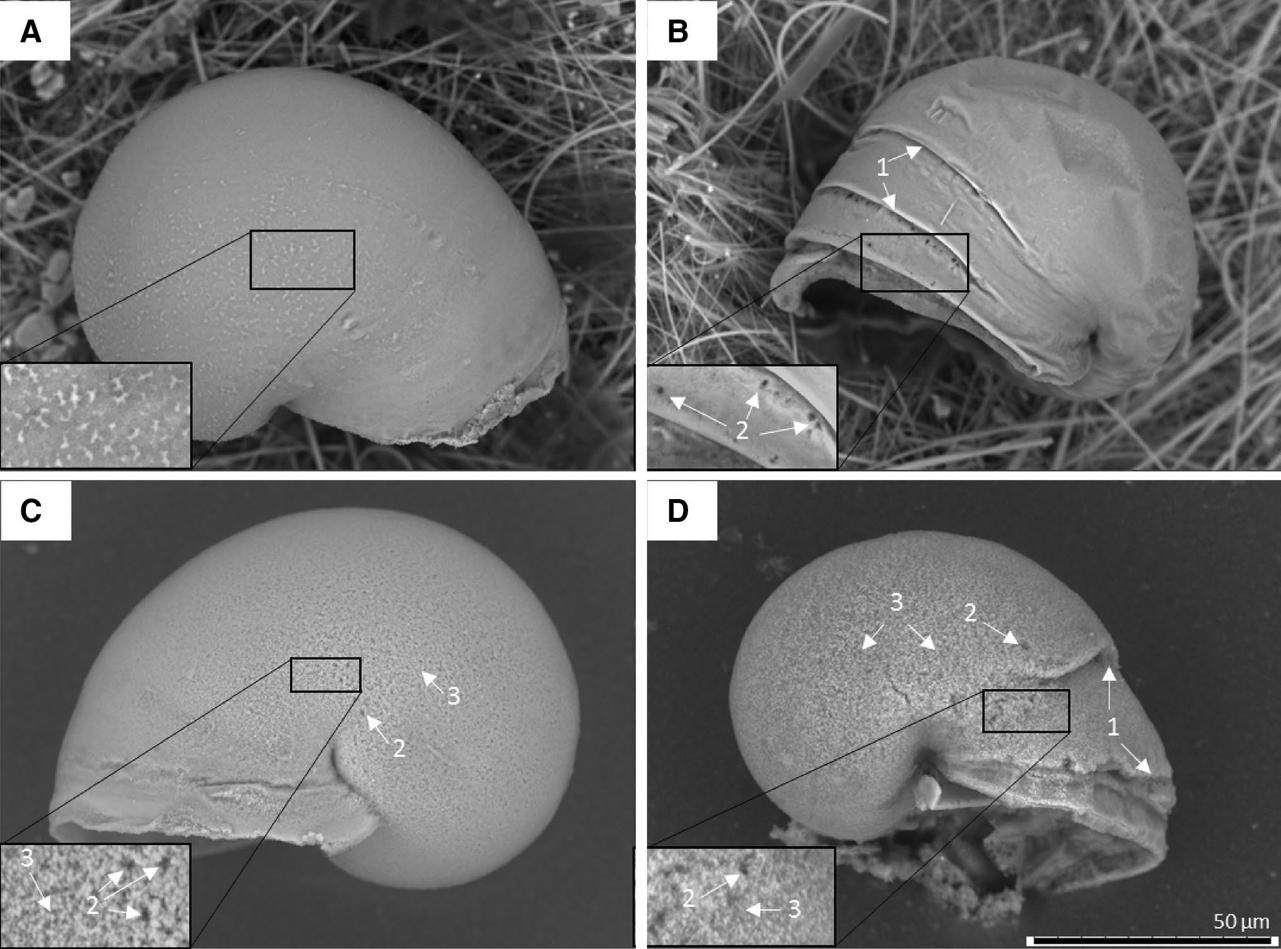
*Limacina helicina antarctica* larval shell morphology as a result of 5 days of exposure to **a** ambient, **b** warm, **c** acidified, and **d** acidified-warm conditions. Examples of malformation (1), pitting (2) and etching (3) are highlighted with arrows. Larvae were alive upon harvesting

Those individuals that exhibited etching and malformations together without pitting most frequently occurred within acidified-warm conditions (87%, *n* = 34), with 10% (*n* = 4) in acidified and 3% (*n* = 1) in warm conditions. A similar number of larvae developed malformation and pitting without etching within warm and acidified-warm conditions at 45% (*n* = 12) and 55% (*n* = 10), respectively. Larvae developed both shell etching and pitting without malformations within acidified-warm (66%, *n* = 19) and acidified (35%, *n* = 10) conditions only. All the larvae that displayed shell pitting, malformation, and etching together occurred within acidified-warm conditions (Fig. 3). The SEM images in Fig. 4 highlight these general combinations of shell morphology.

## Discussion

### Ocean acidification increases larval mortality

We demonstrate that veligers of *L. helicina antarctica* are sensitive to warm, acidified, and acidified-warm oceanic conditions predicted for 2100 in the Scotia Sea (IPCC 2013; McNeil and Matear 2008), given that there was a high level of larval mortality on exposure to these conditions. The previous studies on Arctic juvenile and adult pteropods incubated in acidified conditions (over 5 and 8 days–1 month) found survivorship of 80–100% (Comeau et al. 2009; Manno et al. 2012; Lischka and Riebesell 2012). Here, we show lower survival (down to 61%) of larval *L. helicina antarctica*. A similar low survivorship was also found on incubation of larval *L. retroversa* in acidified conditions indicating increased sensitivity of early life stages (Thabet et al. 2015). This fits the general trend that the early stage molluscs are more vulnerable to acidification than adults (Kroeker et al. 2013; Waldbusser et al. 2015a). Acidification rather than warming appears to be the main driver of increased mortality. Furthermore, there was no synergistic or additive increase in mortality through the addition of warming to acidification. Acidification, therefore, poses the greatest threat to survivorship of larval *L. helicina antarctica.* However, warming has other sub-lethal influences on shell production and maintenance that may increase vulnerability in the natural environment. Interestingly, mortality did not change with exposure time, suggesting that either fatalities were sensitive phenotypes that would have died regardless of the exposure timeframe or because larvae were more sensitive in the first day of exposure and the more vulnerable died sooner. The short time-scale over which these effects were observed has particular relevance to the environmental experience of pteropods, which are most likely to be exposed to such conditions through contact with mesoscale bodies of water where such altered conditions prevail (Bednaršek et al. 2012a). In the Southern Ocean, pH exhibits spatiotemporal variation with water masses, meltwater, season, and phytoplankton productivity (Kapsenberg et al. 2015; Schram et al. 2015). Furthermore, the continued uptake of anthropogenic CO_2_ by the surface ocean is predicted to make undersaturation events occur more frequently over the coming decades where water bodies may become corrosive to aragonite during wintertime by 2038 and will be widespread across the Southern Ocean by 2100 (McNeil and Matear 2008).

### Ocean acidification and warming decreases shell size

For shell growth to occur, larvae take up carbonate ions from the surrounding seawater and concentrate them within the isolated extrapallial space. With acidification, the concentration of surrounding carbonate ions declines and, therefore, more energy is needed for calcification. Larval shell size increased in all treatments, even when exposed to acidified and warm conditions. Continuing shell calcification, despite exposure to acidified conditions and *Ω*_ar_ < 1, has also be observed in Arctic *L. retroversa* (Manno et al. 2012) and *L. helicina helicina* (Lischka et al. 2011; Comeau et al. 2010b). Previously, Comeau et al. (2009, 2010b) demonstrated that calcification stopped when the saturation state of aragonite was below 0.7 in Arctic *L. helicina helicina.* Larvae in the present study were incubated at *Ω*_ar_ = 0.62 and 0.61 in acidified and acidified-warm conditions, respectively, and shell growth continued. This suggests some resilience of these early life stages to short-term exposure to OA (Lischka et al. 2011; Comeau et al. 2010a); however, shell morphology was still altered. Larval shell sizes were smaller upon exposure to acidified and acidified-warm conditions than in ambient conditions. Smaller shell size with exposure to acidification has also been observed in *L. helicina* juveniles and adults (Comeau et al. 2009; Lischka et al. 2011; Comeau et al. 2012). We found that warming and acidification did not interact and further impact larval shell growth, indicating that warming did not mitigate the impact of OA.

Decreased shell size and delayed growth can be attributed to impeded shell deposition, dissolution exceeding calcification, and reduced energetic capacity (Watson et al. 2009). Since larval shell growth increased on exposure to acidified conditions initially and shell etching was observed throughout the 5-day period, it is unlikely that the mechanistic capacity of shell formation was exceeded. However, increased acid–base regulation in acidified conditions is energetically demanding and may explain smaller shell sizes. Altering external conditions increases the energetic demand of maintaining homeostasis and where these costs cannot be met; complete or partial metabolic suppression may be induced as an adaptive strategy to extend survival time (Pörtner 2008).

Juvenile *L. helicina antarctica* exposed to acidic conditions suppressed their metabolic rate (Seibel et al. 2012), while Arctic *L. helicina helicina* exposed to acidified-warm conditions increased their metabolic rate (Lischka and Riebesell 2016). Altering metabolic rate enables energetic allocation to essential physiological processes at the expense of other processes, including shell formation (Pörtner 2008). Food availability has been shown to mediate the impact of ocean acidification in calcifying organisms; therefore, it is possible that with food acquisition, the impacts observed within this study could decline (Seibel et al. 2012; Ramajo et al. 2016). *L. helicina antarctica* veligers are able to feed directly after hatching and are, therefore, probably not dependent on egg stores (Paranjape 1968; Böer et al. 2005). Current estimations vary in whether *L. helicina antarctica* overwinter as larvae, when food availability is naturally low or in the summer, when it is high (Hunt et al. 2008; Bednaršek et al. 2012b; Wang et al. 2017). Since shell growth reduced by day 3 and ceased by day 4 in all treatments, we hypothesise that here, larvae may have been initially utilising endogenous reserves and that these were depleted at differing rates between treatments depending on the energetic cost of maintaining homeostasis, finally inducing a stasis in shell size.

### Shell morphology is altered in a high CO_2_ world

We show that larvae incubated under acidified-warm conditions displayed a combination of both shell etching and shell malformation. The previous studies suggested that warming may offset the negative impacts of ocean acidification (sea urchin: Brennand et al. 2010, coral: McCulloch et al. 2012), although others have revealed cumulative (diatom: Boyd et al. 2015) and even synergistic interactions (pteropod: Lischka and Riebesell 2012). Here, we demonstrate that the impacts of warming and acidification on larval shell morphology are separate, with warming initiating shell malformations and acidification resulting in shell etching. This lack of interaction between warming and acidification has also been observed in Arctic *L. helicina helicina* and *L. retroversa* juveniles (Lischka et al. 2011; Comeau et al. 2010b; Lischka and Riebesell 2012). It suggests that warming and acidification impact different metabolic processes, thus resulting in malformation and etching, respectively, in pteropod larvae.

Temperature has been shown to have a significant effect on biomineralisation processes and growth across a number of calcifying species (Gazeau et al. 2013). Increased temperature can boost shell growth within an organism’s thermal tolerance window and aid acclimatisation to warming (Somero 2010), but shell microstructure can be altered when this optimum is exceeded (Mackenzie et al. 2014), resulting in shell malformations as observed in the current study (Fig. 1). Regions of strain on a shell, such as areas of attachment, are particularly susceptible to disruption which could explain the banding of malformations occurring parallel to the aperture observed in the current study. Since pitting and malformation occurred in warm and acidified-warm conditions, this suggests that pitting is a result of malformation, perhaps as points of failure where shells surpass their physical limits and causing deformation. Acidification was also shown to cause shell pitting, which is consistent with prior studies (Auzoux-Bordenave et al. 2010; Bednaršek et al. 2012b). Shell pitting can, therefore, occur as a result of two different processes and could signify exposure to acidification, warming, or both. Overall, this demonstrates the structural fragility and loss of integrity of larval shells in high CO_2_ conditions.

In contrast to warming, the ability of larvae to counteract acidification depends on a combination of the energetic capability to repair shell damage internally (Lischka et al. 2011; Waldbusser et al. 2013, 2015b), the effectiveness and intactness of the protective organic matrix (periostracum) surrounding the shell (Peck et al. 2016a, b), and the ability to regulate ion and acid–base balance to maintain pH at the site of calcification (Thorp and Covich 2009). On exposure of *L. helicina antarctica* larvae to acidified conditions, shell etching occurred in 100% of individuals from day 1, suggesting a failure in one of these mechanisms. This percentage is higher than previously observed in later life stages (Bednaršek et al. 2012b, 2014; Seibel et al. 2012), indicating that *L. helicina antarctica* larvae may be particularly sensitive to shell etching, although direct comparison is difficult due to variation in species, origin, and methodology (Gazeau et al. 2013). Many early life history stages of gastropods lack specialised ion-regulatory mechanisms required for acid–base maintenance (Ries 2011a, b). However, since shell size continued to increase and etching occurred on the upper shell surface, it is unlikely that this was the main cause of shell dissolution. Numerous early larval stages of benthic gastropods secrete amorphous calcium carbonate, which is more prone to dissolution, before a transition to aragonite (Weiss et al. 2002; Melzner et al. 2011; Duquette et al. 2017). If this was true for pteropods, it would explain why the protoconches of arctic *L. helicina helicina*, that represent larval shells, are particularly susceptible to shell dissolution compared to outer whorls formed in later life stages (Peck et al. 2016a, b). Regardless of the shell composition, the periostracum may have been breached, ineffective, or absent for etching to have occurred (Peck et al. 2016a, b), although the exact role of a pteropod’s periostracum as protection against ocean acidification requires further investigation (Ries 2011a, b; Bednaršek et al. 2016a, b). Since etching did not occur in patterns indicative of abrasion or cracking and there was no mechanism for this to occur, it is unlikely that the periostacum was breached. Furthermore, a mechanism allowing isolation of the extrapallial space from the surrounding undersaturated seawater is needed for calcification to proceed, suggesting that a periostracum is present. We suggest that the periostracum is not as developed in the newly hatched larvae as in later life stages of pteropods and is, therefore, inadequate in protecting larval shells from acidification.

The capacity of pteropods to maintain a viable population distribution and abundance in the Southern Ocean depends on their capability to recruit successfully. We showed that OA and warming do not act synergistically, with the nature of the impacts on viability being recognisably different between the two. Survivorship was mainly influenced by the level of acidification, while the effects of warming were more likely to be sub-lethal and did not increase mortality levels when combined with acidification. We demonstrate that the short-term exposures that are likely to be experienced in the natural environment of larval *L. helicina antarctica* will have a major impact on survivorship and consequently, population stability in these regions.

## Electronic supplementary material

Below is the link to the electronic supplementary material.

Supplementary material 1 (PDF 521 kb)
