# Supplementary material for: Southern Ocean pteropods at risk from ocean warming and acidification
Source: Mar Biol. 2017 Nov 10;165(1):8. doi: 10.1007/s00227-017-3261-3 (PMC5681611; doi:10.1007/s00227-017-3261-3)
Supplement: Supplementary file 1 — Supplementary material 1 (PDF 521 kb) [file 227_2017_3261_MOESM1_ESM.pdf]

Supplementary material for: **Southern Ocean pteropods at risk from ocean warming and acidification.**

Jessie Gardner[1, 2*], Clara Manno[1, 2], Dorothee C. E. Bakker[2], Victoria L. Peck[1] and Geraint A. Tarling[1, 2]

[1]British Antarctic Survey, Natural Environment Research Council, Cambridge, UK.

[2]Centre for Ocean and Atmospheric Sciences, School of Environmental Sciences, University of East Anglia, Norwich, UK.

Table 1. Binomial (logit) generalised linear model comparing mortalities of larval *Limacina helicina antarctica* within control, ambient, acidified and acidified-warm conditions over time (days) using 300 observations with 297.45 null deviance.

**Predictor**	**Estimate (S.E.)**	**Wald**	***p***
Intercept	-3.47 (0.62)	-5.59	<0.001
Warm	1.13 (0.63)	1.81	0.07
Acidified	2.69 (0.58)	4.63	<0.001
Acidified-Warm	2.07 (0.59)	3.49	<0.001
Day 2	0.08 (0.48)	0.15	0.88
Day 3	0.42 (0.47)	0.91	0.36
Day 4	0.73 (0.46)	1.60	0.11
Day 5	0.20 (0.48)	0.41	0.68

Table 2. Binomial (logit) generalised linear model using a two-way factorial design estimating mortality of larval *Limacina helicina antarctica* within control, ambient, acidified and acidified-warm conditions over time (days) using 300 observations with 297.45 null deviance.

**Predictor**	**Estimate (S.E.)**	**Wald**	***p***
Intercept	-3.68 (0.89)	-4.12	<0.001
Warm	0.92 (0.86)	1.06	0.28
Acidified	3.01 (0.89)	3.38	<0.001
Day	0.15 (0.23)	0.69	0.49
Acidified:Warm	-1.67 (0.70)	-2.38	0.02
Acidified:Day	-0.10 (0.22)	-0.46	0.64
Warming:Day	0.062 (0.21)	0.30	0.76

Table 3. Summary of larval *Limacina helicina antarctica* mortalities/breakages within each treatment and across exposure times. These individuals were not considered for further analysis. Overall 223 larvae were analysed for changes in shell morphology.

		Number of deaths			
		**Ambient**	**Warm**	**Acidified**	**Acidified-Warm**
Exposure time (days)	**1**	0/0	0/6	6/1	3/1
	**2**	0/3	2/1	5/0	4/0
	**3**	1/1	2/0	6/1	4/0
	**4**	1/4	2/1	6/1	4/2
	**5**	0/1	2/0	5/0	3/0

Table 4. Gamma (identity) maximum likelihood generalised linear comparing shell size of larval *Limacina helicina antarctica* within control, ambient, acidified and acidified-warm conditions over time (days) using 223 observations with 3.38 null deviance.

**Predictor**	**Estimate (S.E.)**	***t***	***p***
Intercept	81.80 (0.73)	111.31	<0.001
Warm	0.98 (1.21)	0.81	0.42
Acidified	-2.92 (1.21)	-2.4	0.01
Acidified-Warm	-5.8 (1.08)	-5.34	<0.001
Day 2	8.2 (1.17)	7.04	<0.001
Day 3	14.77 (1.16)	12.72	<0.001
Day 4	29.02 (1.38)	22.1	<0.001
Day 5	31.2 (1.28)	24.33	<0.001
Acidified-Warm: Day 2	-4.29 (1.64)	-2.61	0.009
Acidified: Day 2	-0.37 (1.79)	-0.21	0.834
Warm: Day 2	-1.39 (1.76)	-0.79	0.428
Acidified-Warm: Day 3	0.86 (1.70)	0.51	0.612
Acidified: Day 3	-0.52 (1.89)	-0.28	0.783
Warm: Day 3	-1.31 (1.76)	-0.74	0.458
Acidified-Warm: Day 4	0.09 (2.03)	0.05	0.963
Acidified: Day 4	-3.02 (2.12)	-1.42	0.16
Warm: Day 4	-7.21 (1.97)	-3.64	<0.001
Acidified-Warm: Day 5	-2.03 (1.84)	-1.10	0.271
Acidified: Day 5	-4.07 (1.98)	-2.05	0.041
Warm: Day 5	-9.43 (1.89)	-4.98	<0.001

Table 5. Gamma (identity) generalised linear model fit by using a two-way factorial design estimating the impact on shell size of exposing larval *Limacina helicina antarctica* to control, ambient, acidified and acidified warm conditions to fit 223 observations with 3.38 null deviance.

**Predictor**	**Estimate (S.E.)**	***t***	***P***
Intercept	75.33 (0.97)	77.54	<0.001
Warm	0.07 (1.29)	0.05	0.95
Acidified	-6.03 (1.29)	-4.67	<0.001
Day	7.55 (0.32)	23.96	<0.001
Acidified:Warm	-0.76 (1.08)	-0.70	0.48
Acid:Day	0.72 (0.38)	1.88	0.061
Warm:Day	-0.79 (0.38)	-2.06	0.039

Table 6. Binomial (logit) generalised linear model estimating the presence of malformation, pitting and etching on larval *Limacina helicina antarctica* shells incubated within control, ambient, acidified and acidified-warm conditions over time (days) using 223 observations with 277.5, 251.35, 307.16 null deviance respectively

Variable	Predictor	Estimate (S.E.)	Wald	*p*
**Malformation**	Intercept	-6.75 (0.94)	-7.15	<0.001
	Warm	3.72 (0.69)	5.35	<0.001
	Acidified	0.72 (0.76)	0.96	0.34
	Acidified-Warm	5.16 (0.79)	6.56	<0.001
	Day 2	1.08 (0.77)	1.41	0.16
	Day 3	2.94 (0.77)	3.82	<0.001
	Day 4	4.71 (0.87)	5.41	<0.001
	Day 5	4.42 (0.84)	5.29	<0.001
**Pitting**	Intercept	-3.86 (0.89)	-4.32	<0.001
	Warm	3.96 (0.88)	4.48	<0.001
	Acidified	2.84 (0.90)	3.13	<0.001
	Acidified-Warm	3.49 (0.89)	3.93	<0.001
	Day 2	0.04 (0.48)	0.09	0.93
	Day 3	-0.22 (0.49)	0.09	0.67
	Day 4	-0.18 (0.51)	-0.36	0.72
	Day 5	-1.03 (0.53)	-1.92	0.05
**Etching**	Intercept	-5.84 (1.59)	-3.67	<0.001
	Warm	1.66 (1.20)	1.38	0.17
	Acidified	11.55 (2.78)	4.16	<0.001
	Acidified-Warm	11.74 (2.79)	4.21	<0.001
	Day 2	-0.32 (1.59)	-0.19	0.84
	Day 3	2.56 (1.37)	1.87	0.06
	Day 4	-0.35 (1.62)	-0.22	0.83
	Day 5	-0.34 (1.57)	-0.22	0.83

Table 7. Binomial (logit) generalised linear model using a two-way factorial design estimating the presence of malformation, pitting and etching on larval *Limacina helicina antarctica* shells incubated within control, ambient, acidified and acidified-warm conditions over time (days) using 223 observations with 277.5, 251.35, 307.16 null deviance respectively.

**Variable**	**Predictor**	**Estimate (S.E.)**	**Wald**	***p***
**Malformation**	Intercept	-13.53 (3.19)	-4.24	<0.001
	Warm	6.82 (1.75)	3.91	<0.001
	Acidified	1.47 (1.26)	1.16	0.24
	Acidified-Warm	0.92 (1.38)	0.67	0.50
	Day	2.17 (0.59)	3.67	<0.001
**Pitting**	Intercept	-2.09 (0.79)	-2.64	<0.01
	Warm	2.21 (0.80)	2.76	<0.01
	Acidified	2.23 (0.79)	2.82	<0.01
	Day	-0.28 (0.22)	-1.29	0.19
	Acidified:Warm	-2.36 (0.79)	-2.98	<0.01
	Acid:Day	-0.04 (0.16)	-0.27	0.78
	Warm:Day	0.20 (0.19)	1.06	0.28
**Etching**	Intercept	-4.49 (1.70)	-2.63	<0.01
	Warm	1.32 (1.72)	0.77	0.44
	Acidified	10.11 (3.02)	3.35	<0.001
	Day	-0.24 (0.52)	-0.45	0.65
	Acidified:Warm	0.11 (2.27)	0.04	0.96
	Acid:Day	0.24 (0.62)	0.38	0.70
	Warm:Day	0.29 (0.57)	0.49	0.62

Table 8. Model selection parameters (residual deviance, residual degrees of freedom, delta AIC and AIC weight) for the top models (delta<4) predicting the effect of acidification and warming over time (days) on *Limacina helicina antarctica* mortality, shell morphology and shell size. Generalised linear model

Variable	Predictor	R. Df	R. Dev	dAIC	Weight
**Mortality**	Acidified *Warm	296	258.96	0.0	0.41
	Acidified*Warm + Day	295	257.77	0.8	0.28
	Acidified*Warm + Day + Acidified:Day	294	257.05	2.1	0.15
	Acidified*Warm + Warm:Day + Acidified	294	257.73	2.8	0.10
	Acidified	298	266.73	3.8	0.06
**Pitting**	Acidified*Warm + Day	218	196.63	0.0	0.27
	Acidified*Warm + Day + Warm:Day	217	194.84	0.2	0.25
	Acidified*Warm + Day + Acidified:Day	217	195.97	1.3	0.14
	Acidified*Warm	219	200.93	2.3	0.09
	Acidified*Day + Warm + Warm:Day	217	194.83	0.2	0.25
**Malformation**	Acidified * Day + Warm	218	128.36	0.3	0.3
	Acidified + Warm + Day	219	130.07	0.0	0.0
	Acidified*Warm + Day	218	130.08	2.0	2.0
	Warm* Day + Acidified	218	129.97	1.9	1.9
	Acidified*Warm + Day + Acidified:Day	217	127.85	1.8	1.8
	Acidified * Day + Warm + Warm:Day	217	128.62	2.6	2.6
**Etching**	Acidified + Warm	220	25.04	0.0	0.43
	Acidified	221	28.68	1.6	0.19
	Warm + Acidified + Day	219	25.02	2.0	0.16
	Acidified*Warm	219	25.05	2.0	0.16
	Acidified + Day	220	28.68	3.6	0.07
**Size**	Acidified*Warm*Day	215	0.36	0.0	1

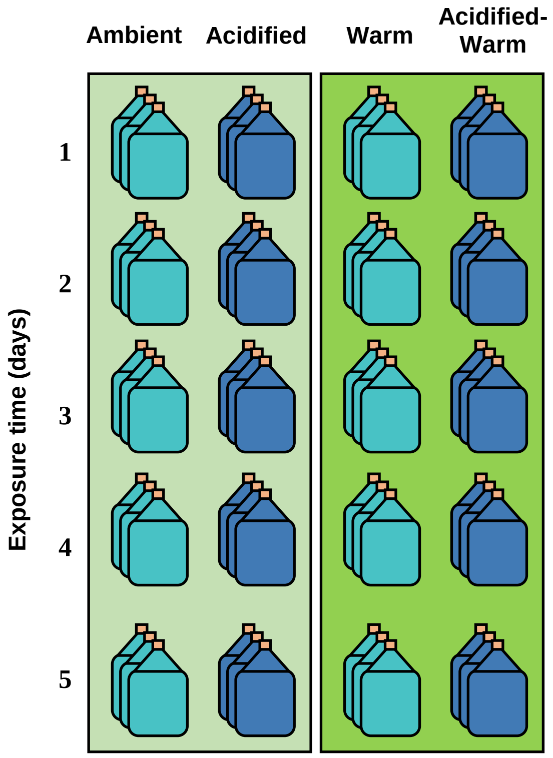

Figure 1. Schematic of the experimental design. A fully factorial design was adopted with 4 treatments representing ambient (1.7 °C, pH 8.1), warm (3.5 °C, pH 8.1), acidified (1.7 °C, pH 7.6) and acidified-warm (3.5 °C, pH 7.6) conditions. Five pteropods were placed in each bottle with three bottles being removed from each treatment every day for five days.

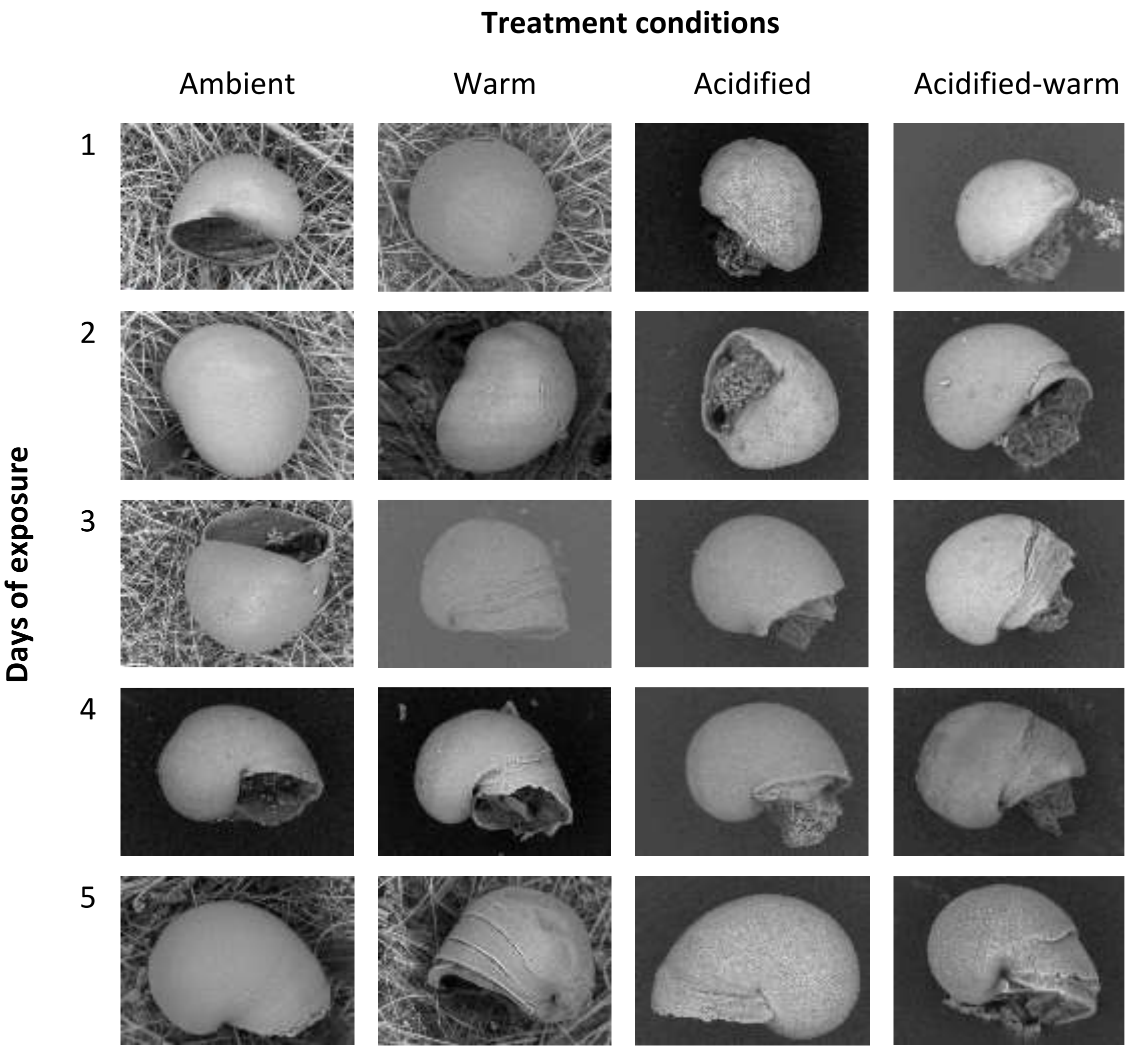

Figure 2. Scanning Electron microscope images of larval *Limacina helicina antarctica* after exposure to ambient, warm, acidified and acidified-warm conditions for 1-5 days. All larvae were alive upon harvesting.
